# The Adipose Tissue at the Crosstalk Between EDCs and Cancer Development

**DOI:** 10.3389/fendo.2021.691658

**Published:** 2021-07-20

**Authors:** Emma Bokobza, Charlotte Hinault, Victor Tiroille, Stéphan Clavel, Frédéric Bost, Nicolas Chevalier

**Affiliations:** ^1^ Université Côte d’Azur, INSERM U1065, C3M, Nice, France; ^2^ Université Côte d’Azur, CHU, INSERM U1065, C3M, Nice, France

**Keywords:** adipose tissue, endocrine disruptor (EDC), cancer, secretome, model, endocrine disrupting chemicals

## Abstract

Obesity is a major public health concern at the origin of many pathologies, including cancers. Among them, the incidence of gastro-intestinal tract cancers is significantly increased, as well as the one of hormone-dependent cancers. The metabolic changes caused by overweight mainly with the development of adipose tissue (AT), insulin resistance and chronic inflammation induce hormonal and/or growth factor imbalances, which impact cell proliferation and differentiation. AT is now considered as the main internal source of endocrine disrupting chemicals (EDCs) representing a low level systemic chronic exposure. Some EDCs are non-metabolizable and can accumulate in AT for a long time. We are chronically exposed to low doses of EDCs able to interfere with the endocrine metabolism of the body. Importantly, several EDCs have been involved in the genesis of obesity affecting profoundly the physiology of AT. In parallel, EDCs have been implicated in the development of cancers, in particular hormone-dependent cancers (prostate, testis, breast, endometrium, thyroid). While it is now well established that AT secretes adipocytokines that promote tumor progression, it is less clear whether they can initiate cancer. Therefore, it is important to better understand the effects of EDCs, and to investigate the buffering effect of AT in the context of progression but also initiation of cancer cells using adequate models recommended to uncover and validate these mechanisms for humans. We will review and argument here the potential role of AT as a crosstalk between EDCs and hormone-dependent cancer development, and how to assess it.

## Introduction

Obesity, defined as an abnormal or excessive fat accumulation in the body, is a major public health concern with more than 650 million obese adults in 2016 (from World Health Organization). Obesity is at the root of many pathologies, whether functional (orthopedic, esthetic, psychological complications, benign (diabetes, endometriosis) or malignant (gastro-intestinal tract and hormone-dependent cancers). Numerous studies demonstrated the relationship between obesity and an increased risk of cancer (1–4). For example, obesity increases the risk of breast cancer after menopause by 8% and is responsible for 34.1% of endometrial cancers. Globally, 15-20% of total cancer-related mortality in adults aged 30 and over are attributable to obesity or overweight. Obesity provokes metabolic changes related to adipose tissue (AT) development such as insulin resistance and chronic inflammation. These changes induce hormonal and/or growth factor imbalances, which impact cell proliferation and differentiation and can also explain the increased risk of obesity-related cancers.

Climate transition which has begun several years ago is associated with the use of many pollutants. Some of these pollutants are chemicals that accumulate during the food chain in different tissues but mainly in adipose tissue (AT) due to their lipophilic nature. Some of these persistent organic pollutants (POPs) have already been regulated and/or withdrawn from the market due to their carcinogenic properties. They are still found in many products from the chemical industry, such as pesticides, some plastics or cleaning products, or even in building materials. They are usually classified into five categories (5): dioxins, polychlorinated biphenyls (PCBs), organochlorine pesticides (OCs), polybrominated flame retardants (PBDE) and perfluorinated compounds (like PFOS and PFOA) found especially in non-stick coatings (6, 7).

Because of their long half-life and their ability to store in AT, populations remain exposed, from fetal life, and therefore at risk of developing pathologies even when they are exposed to low doses of these pollutants. It has been shown that some of these POPs could interfere with hormonal signaling and/or regulation pathways, thus giving them endocrine disrupting (ED) activity (5). Early and/or chronic exposure to POPs with ED properties can modify the incidence of certain diseases, in particular obesity (8) or hormone-sensitive cancers (thyroid, prostate, testis, breast, ovary) (9, 10).

Hence, there are credible to convincing evidence for the link between obesity and cancer in one hand, and on the other hand, as we will discuss below, between ED chemicals (EDCs) and obesity or EDCs and cancer. However, the link between EDCs, obesity and cancer have not been yet demonstrated. This link could be the adipose secretome perturbed by EDCs, which modify the balance between proliferation and differentiation cell processes.

## EDCs and Hormone Sensitive Cancers

The development of some cancers is stimulated by hormones, which naturally circulate in the body and bind to membrane and/or nuclear receptors of cancer cells favoring their growth and multiplication. Among these hormone-dependent cancers, prostate cancer (PCa) and endometrial cancer (ECa) are the most common cancers of the male and female reproductive systems, respectively, in addition to breast cancer (BCa) which is the most common cancer in women worldwide (2). Steroid hormones (estrogens, androgens) play an important role in the etiology, progression and treatment of hormone-dependent cancers (11–13). It is therefore obvious that exposure to EDCs can influence the incidence and development of those cancers (9).

EDCs have been firstly identified as risk factor with the dramatic story of diethylstilbestrol (DES) (14). This synthetic diphenol with potent estrogenic properties was widely prescribed to pregnant women until 1970s to reduce the risk of abortion; however, several studies have reported an increased risk of rare cancers in women progenies (14, 15). Importantly, deleterious effects of prenatal DES exposure have been shown to persist in second-generation paving the way of the concepts of epigenetic transgenerational inheritance (16). Since then, several epidemiological studies supported by *in vivo* and *in vitro* experiments have confirmed this association between EDCs (notably PCBs, dioxins, DDE and bisphenol A [BPA]) and an increased risk of hormone-dependent cancers in both sexes (7, 17–19). Regarding testicular cancer, we and others have shown that BPA was able to stimulate the proliferation of seminoma cells involving GPR30/GPER pathway (20–22). Concerning PCa, Prins et al. have shown that exposure to BPA makes prostate stem cells more sensitive to estrogen in adulthood and therefore more likely to develop PCa (23, 24). Regarding persistent EDCs, although discussed, exposure to chlordecone constitutes a demonstrative example with a significant increase in the risk of PCa (25) and of recurrence after radical prostatectomy (26). Observational and experimental studies have suggested a role of PCB-153, an industrial organochlorine product, in the development of high-grade PCa (27). However, a previous study observed an inverse correlation between plasma concentrations of PCB-153 and PCa (28). Likewise, studies differ about a positive association (29) or not (30) between elevated serum levels of PFOA and PCa onset and/or progression. Thus, despite this extensive work on the role of certain EDCs in the incidence of hormone-sensitive cancers, diverse investigations for their action modes, their effects on tumor growth and on the formation of metastases especially in human are still poorly understood (7, 31).

## EDCs and Adipose Tissue

AT is a major player in toxicological responses to exposure to EDCs, especially to POPs with predominantly halogenated structure which makes them non-metabolizable and very lipophilic (32). By storing POPs, AT may appear to have a protective role, but it is rather considered to be the main internal source of chronic low-level systemic exposure to EDCs since they will be released progressively or massively when lipolysis will occur. Therefore, AT represents a dynamic storage compartment for EDCs within the body with a continuous flow between storage and release in post-exposure periods. Various *in vitro* and *in vivo* studies have focused on this dynamic mobilization of EDCs by AT, for instance a murine cell model mimicking lipolysis has been developed and tested for PCBs (33). Using a xenografted fat model, others have shown that TCDD stored in AT of xenograft can be released into the recipient mice and modify gene expression providing a direct evidence of the potential deleterious effects of TCDD (34). Recently, we have shown in a large prospective study that massive weight loss during the first year following bariatric surgery is associated with a prolonged release of POPs from AT, mainly PCB-153, DDE and hexachlorobenzene (35). The fat depot specific differences in EDC bioaccumulation have also been investigating but to date divergent results were obtained even though there is an agreement concerning the abundance of certain EDCs and the correlation with AT macrophage infiltration, adipocyte size or with metabolic parameters (36, 37).

In addition to its storage role, AT functions as a full-fledged endocrine organ producing and responding to hormones and adipokines (38–40). Several EDCs have been described *in vitro* and/or *in vivo* to profoundly affect AT physiology: adipocyte differentiation, adipocytokine secretion, oxidative stress and inflammation (8, 41, 42). Numerous publications have demonstrated a possible role for EDCs in the genesis of obesity, they have been called obesogen based on the hypothesis of Blumberg and Grun (8, 38, 41, 43, 44). Indeed, in case of chronic high caloric intake, AT undergoes into morphological changes: hyperplasia (increase of adipocytes number) and hypertrophy (lipid accumulation in the adipocytes resulting in the increase of adipocyte size) (38, 45). Hyperplasia takes place in healthy AT expansion. However, hypertrophy leads to dysfunctional adipocytes development, secreting adipokines as leptin, adiponectin or resistin for the main ones in addition to pro-inflammatory adipocytokines such as MCP-1, TNF-alpha, IL-6, IL-8. Hypertrophy also increase hypoxia, decreasing vascular supply resulting in adipocyte death by a rupture of the membrane, leading to a release of cellular content into the microenvironment. All of this results in the infiltration of inflammatory immune cells including lymphocytes, granulocytes type 1 macrophages leading to a change in AT microenvironment characterized by a chronic inflammation, the development of crown-like structures (dead adipocytes surrounded by macrophages within AT). Those structures generate reactive oxygen species (ROS) that are likely to induce DNA damages. This low-grade chronic inflammation affects local metabolism, but also systemic energy homeostasis. EDCs can act on hyperplasia and/or hypertrophy of the AT but also on adipose secretion (32, 46). For example, because PPAR is a key molecule in the regulation of adipogenesis, any EDC acting as an agonist on this receptor will be likely to cause an expansion of adipocytes, and therefore a modification of the secretome and act as an obesogenic EDC. It is the case of tributyltin (TBT) (44) and PFOA (29, 47–50). Indeed, TBT has been shown to promote inflammatory infiltration into adipocytes but also in reproductive tract in addition to increase fat mass (51–53). In addition to binding to PPAR, some EDCs promote adipogenesis by other mechanisms, such as *via* estrogen, glucocorticoid receptors or others. It has also been shown that some EDCs, such as dioxins, were able to induce a pro-inflammatory action on murine (54) and human (55) adipocyte cells, as well as TCDD in mouse AT through AhR pathway (55). In parallel to *in vitro* and *in vivo* studies, several epidemiological studies support the association of pre/postnatal exposure to EDCs and increased BMI with the concept of transgenerational effects on progeny (7, 8, 41, 44). Therefore, EDCs that disrupt the coordinated regulation of adipocyte development, metabolism and endocrine function may result in disturbances in local and systemic energy metabolism and inflammatory response (56). The impact of EDCs on adipocyte endocrine function have been investigated but mainly in the context of obesity and/or cardiometabolic disorders. Further studies are required to fully examine their role alone or in cocktail at different doses and exposure notably in the context of cancer.

## Cancer and Adipocyte Secretome

Despite recent advances in understanding the biological basis of cancer, the mechanisms underlying its metastatic spread are not clearly established. In this process, the tumor environment plays an essential role. Indeed, a dialogue between cancer cells, the immune system and neighboring tissue cells such as AT is established and modulates the growth and migration of cancer (57, 58). This tumor microenvironment can also transform some adipocytes in so-called cancer-associated adipocytes (CAA) (59). It is also now well established that AT is a key player in the tumor microenvironment, by secreting factors that promote tumor progression and/or by providing metabolite substrates to cancer cells (38, 60–62). An excessive development of AT, as observed in obesity, associated with the existence of a metabolic syndrome, has been correlated with a marked increase in the aggressiveness of cancers (38, 63–65). Adipocytes and AT cells secretome is composed of lipids, adipokines, inflammatory cytokines, peptide hormones as well as extracellular vesicles working both in paracrine and endocrine, extracellular matrix components (38, 60–62).

Among paracrine and endocrine effects, the best characterized adipocytokines are the leptin and the adiponectin (66, 67). *In vitro* studies have demonstrated that leptin was able to activate ERK1/2 and c-Jun NH2-terminal Kinase (JNK) pathway and so promote cancer cell proliferation (68). However, no strong evidence showed the *in vivo* implication of leptin in tumorigenesis, although leptin levels or leptin signaling dysregulation have been observed in BCa, PCa and ECa (69). Concerning adiponectin, which circulating levels is inversely correlated to obesity, *in vitro* studies have shown its inhibitor role in proliferation and apoptosis in cancer cell line such as liver, breast, endometrium and stomach through the activation of AMPK and the inhibition of PI3K/Akt, ERK1/2 pathway, NF-κB, Wnt-β-catenin pathway (70). Similarly, *in vivo* experiments demonstrated that adiponectin reduced tumorogenesis of cancer cells and that adiponectin-deficient mice developed more tumors (71). Clinical studies indicated a positive correlation between leptin:adiponectin ratio and increased risks for some cancers like ECa in post-menopausal women (72), BCa (73) and PCa (74). However, a metanalysis has then discussed leptin:adiponectin ratio and demonstrated no strong prognosis value for PCa (75).

In addition to adipokines, other factors secreted by adipocytes are involved in tumor progression processes more particularly through a paracrine action. In case of PCa, adipocytes from periprostatic AT (PPAT) secrete CC-chemokine ligand 7 (CCL7) which can diffuse through prostatic capsule to reach the tumor. Interaction between CCL7 and its ligand CC-chemokine receptor 3 (CCR3) will allow tumor migration outside of prostatic gland and initiate metastatic process (61, 76). More recently, creatine has been identified as metabolic substrate in BCa cells (77), which accelerates tumor progression due to a transformation into phosphocreatine to fuel tumor growth, especially in the context of obesity (77). In mammary human tumor, it has been shown that adipocytes in contact with the tumor presented phenotypic modification such as delipidation, dedifferentiation, with an overexpression of pro-inflammatory cytokines such as IL-6 (78).

The bidirectional communication between tumors and adipocytes have also been shown. After invading AT, tumors induce adipocyte lipolysis and thereby released fatty acids, stimulate ROS production favoring tumor invasion (79). Moreover, some AT cells called adipose stromal cells (ASC, multipotent mesenchymal progenitors) can be recruited from AT tumor through chemokine gradient (62) and then enhance PCa progression (80). Besides, FABP4, an abundant adipocyte protein, has been shown to be secreted by adipocytes but also by PCa and stromal cells. This put FABP4 at the heart of a communication between adipocytes and tumor stimulating MMPs and cytokine production in the PCa stromal microenvironment to favor tumor progression (81). More examples of transmitting signals between tumor cells and adipocytes including the potential implication of extravesicles/exosomes have been reviewed elsewhere (39, 57).

**Figure 1:**
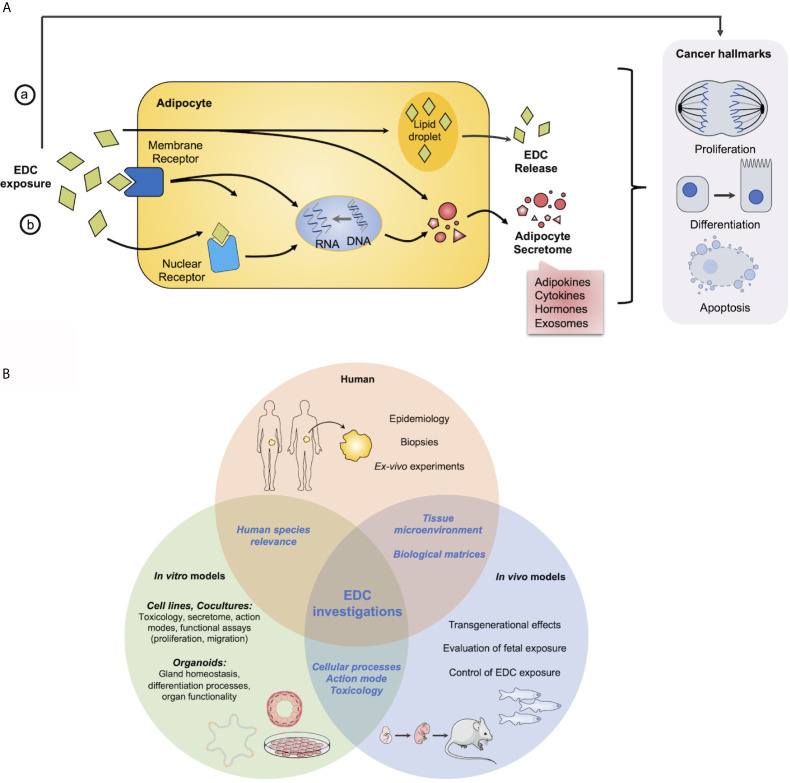
Overview of EDCs action in adipocytes and cancer cells and models to study EDCs. **(A)** Potential or hypothetical model of how EDCs can interfere in hormone sensitive cancer development or progression with adipocytes. EDCs can act a) directly on hormone-sensitive cells to modify their physiology and/or function to induce or exacerbate cancer hallmarks and/or b) on adipocytes by interfering with (i) nuclear or (ii) membrane receptors to modulate adipocyte secretome through genomic or non-genomic pathways, otherwise EDCs can be stored in (iii) lipid droplets and be released, progressively during all the life or massively as observed after a weight lost. **(B)** Requirement of complementary approaches to investigate impact of EDCs on health. Main questions and assays from *in vitro* and *in vivo* models to human.

Overall, obesity represents a high-risk factor for several cancers because it promotes AT remodeling which can favor tumorogenesis and tumor progression through a crosstalk between tumor cells and adipocytes. Adipocyte secretome has been so far rather implicated in aggressiveness than in initiation of tumorogenesis. These deleterious effects of AT on cancer cells could be induced or exacerbated by the POPs stored there, which could therefore play an important role in the initiation, progression and/or metastasis of hormone-sensitive cancers which develop later in life (**Figure 1A**).

## Models to Assess EDCs Effect on Adipose Secretome and Cancer Development

Data from epidemiological studies are essential for the detection of potential adverse effects of EDCs but usually provide only suggestive data (41). However, regulators need strong proofs of the interferences of EDCs with the hormonal system. Thus, *in vitro* assays are required to decipher EDCs molecular mechanisms. To validate *in vitro* experiments and implement them with physiological and transgenerational data, *in vivo* or at least *ex-vivo* models are needed. Thus, it is important to develop alternative systems making it possible to screen these molecules, upstream, to demonstrate in a reliable, reproducible and robust manner, their safety or their potential toxicity **(** **Table 1** **)**. Multiple alternative systems have been developed with frog embryos as developmental toxicity test or zebrafish xenograft assay (5).

**Table 1:** Overview of in vivo and in vitro models available to study the adverse effect of EDCs.

		Epidemiology	EDC Storage/Release	EDC Signaling	Cell Death	Cell Proliferation	Cell Differentiation	Inter/Transgenerational effects
***In vivo***	**Human samples**	+++	++	–	–	–	–	+
	**Animal models**							
	Rodent							
	Xenope	–	+++	+	+	+	++	+++
	Zebrafish							
***In vitro***	**Organoid models**							
	Thyroid							
	Breast							
	Prostate	–	-	++	+++	+++	+++	–
	Endometrium							
	**Cancer cell lines**	–	–	+++	+++	+++	–	–
	**Pre/Adipocyte cultures**	–	+++	+++	–	+	+++	–

-/+++: low to high relevant models to assess the effects of EDCs on adipose secretome and cancer development.

Regarding adipose models, 3T3-L1 mouse cells is the most used cell line with the subclone 3T3-F442A. They have allowed to decipher adipogenesis molecular mechanisms and regulations and to screen multiple drugs before starting clinical trials (82). EDCs have been extensively studied in these cells notably to examine their obesogenic capacity (8, 41, 43, 44). To understand the impact of adipocyte secretome, coculture have been developed. Initially, indirect cocultures were performed by incubating cells with adipocyte conditioned medium. This approach has been conducted with different cell types such as melanoma (83), glioma (84) or PCa cells (76). 3T3L-1 as well as *ex-vivo* AT conditioned medium were able to increase overall survival of cancer cell lines both by increasing proliferation and decreasing apoptosis (83, 84) and to promote migration of tumor cells (76). Based on these approaches and to understand the potential role of EDCs on tumorogenesis or tumor progression through the modification of AT secretome, normal or tumoral cell lines/primary cells of an organ could be incubated with conditioned media of adipocytes prior exposed to EDCs. Another way to study adipocyte secretome is to perform “direct” coculture assay based on Boyden chambers with an insert. For instance, cocultures of BCa and 3T3-F442A cells were used to study the bidirectionel communication between these cells (78, 79).

The results obtained from toxicity and toxicokinetic studies conducted on animals are usually difficult to transpose to humans (41). Over the past years, human multipotent cell models have been developed, notably hMADS cells (human multipotent adipose-derived stem cells) (85) and hASCs (human primary adipose‐derived stromal/stem cells) (86), which allow to well-characterize the different events of lineage commitment (82). TCDD has been shown to increase inflammatory gene expression in hMADS cells but more strongly in undifferentiated than in differentiated adipocytes (55). Bisphenol S has been shown to deregulates adipokine secretion in a fat depot-specific manner in omental *versus* subcutaneous derived adipocytes from hASCs (87). More recently, Koual et al. have shown that coculture of human BCa cells with hMADS cells, although not differentiated into mature adipocytes, treated with TCDD leads to an increased MCF7 cell growth (19).

While 2D cultures of ASCs are easy to isolate and to differentiate into mature adipocytes, they present numerous limitations, including immortalization and lack of neighboring cells. ASCs have been shown to contribute to AT microenvironment given the opportunity to develop *in vitro* tissue-engineered adipose models such as 3D culture and/or cocultures with other cell types (i.e. endothelial cells or macrophages) (88–92). Recently, self-assembled adipose constructs into 3D spheroids using primary human SVF cells and a human blood product-derived biological scaffold have been validated (91). 3D adipocyte cultures bring new insight to study connective tissue interactions and crosstalk with other cells such as cancer cells. Using adipocytes in a 3D collagen gel matrix, proliferation rate of human bone-metastatic PCa cell line PC3 increased as well as the expression of VEGF and PDGF (93), as it was previously observed with LNCaP or DU145 cells (94).

The other main limitation of classical cell culture is the use of only one cell type while an organ is composed of several cell types that communicate together. This issue can be resolved using a cutting-edge technology developed this past decade: *organoids* (95, 96). Organoids allow the study of cell-cell communication but also organ functionality. Organoids have been developed for hormone sensitive organ (thyroid, prostate, testis, endometrium, ovarian); their potential applications and limitations have been recently reviewed (97–100). Furthermore, organoid treated with special drugs presents phenotypic and morphological specificities and this allows an easy and relatively cheap drug-screening platform (101), including the field of precision medicine (102) but also the identification of adverse effects of EDCs as discussed for thyroid gland (98). Therefore, organoids can be a good model to screen EDCs impact on the balance of cell differentiation/proliferation through modification of adipocyte secretome. However, because of medium incompatibility, cocultures using organoids and adipose cells, or direct incubation with adipocyte-conditioned medium cannot be performed, even with immune (103), stromal or vascular components (104). This demonstrates the necessity to developed adapted technology such as microfluidic system as already described in BCa cells (105) or to identify specific molecules present in AT secretome by omics approaches.

Therefore, multiple models have been developed to study EDCs impact on the physiology of diverse tissue/organ, more precisely EDCs storage and release from AT and EDCs effects on proliferation and differentiation of hormone-sensitive cells which are summarized in **Figure 1B**.

## Conclusion

To study the impact of EDCs on tumor initiation/progression, it is important to be able to provide relevant tools allowing predictive analysis, upstream of the health risk, and in particular carcinogenic, of these molecules. There are inherent biases in epidemiological studies considering EDCs, which prevent definitive data on their role in carcinogenesis/metastatic spread. It is currently recognized that chronic exposure to EDCs may be responsible for an over-incidence of hormone-dependent cancers in humans and that EDCs impact on AT functioning. However, the links between EDCs, AT and cancer remain largely unknown. Therefore, alterations in AT secretome by EDCs could allow to identify specific markers, predictive factors of tumor progression, usable for various stakeholders in the field (clinicians, manufacturers, decision-making bodies and regulatory health agencies). A better understanding of the functional alterations in AT by EDCs could therefore provide explanatory avenues to elucidate the complex links between obesity and some types of cancer.

## Author Contributions

EB, CH, and NC wrote the manuscript. VT, SC, and FB contributed to discussions and manuscript. All authors contributed to the article and approved the submitted version.

## Funding

The team was supported by the Institut National de la Santé Et de la Recherche Médicale (INSERM), by the Agence Nationale de SEcurité Sanitaire de l’alimentation, de l’environnement et du travail (ANSES) (PNREST 2017 - IncuPE to Nicolas Chevalier) and the IDEX-UCA^JEDI^ program (AAP3 2018 - ProstAdiPE and 2021- OrgaPEPro to Charlotte Hinault).

## Conflict of Interest

The authors declare that the research was conducted in the absence of any commercial or financial relationships that could be construed as a potential conflict of interest.
